# Estimating ruminal crude protein degradation from beef cattle feedstuff

**DOI:** 10.1038/s41598-019-47768-3

**Published:** 2019-08-06

**Authors:** Chang Liu, Deyong Li, Wanbao Chen, Yan Li, Hao Wu, Qingxiang Meng, Zhenming Zhou

**Affiliations:** State Key Laboratory of Animal Nutrition, College of Animal Science and Technology, China Agricultural University, Beijing, 100193 China

**Keywords:** Behavioural methods, Animal behaviour

## Abstract

We estimated ruminal crude protein degradation of twelve feedstuffs commonly used in China using *in vitro* and *in vivo* methods. The *in vivo* net protein utilization (NPU) levels of corn, sorghum, barley, wheat, Chinese wild rye grass, corn stalk, rice straw, soybean straw, soybean meal, distillers’ dried grains with solubles (DDGS), Brewers’ spent grains, and sunflower meal were 52.57, 49.68, 65.38, 72.58, 82.41, 72.26, 68.57, 76.95, 54.75, 56.27, 29.03 and 41.88%, respectively. The linear regression between NH_3_-N incorporated into microbial proteins and gas production after incubation (6, 12, and 24 h) was significant (r = 0.9948 and P < 0.001, r = 0.9874 and P < 0.01, and r = 0.9912 and P < 0.01, respectively). Based on the linear regression equations, we estimated *in vitro* protein degradability (IVPD) and generated the regression equations between IVPD and NPU. The linear regression equations between IVPD and NPU after 6 h incubation in the energy, protein, and roughage feed groups were Y = 0.5633X + 33.20 (R^2^ = 0.8517, P < 0.05), Y = 0.8482X+ 34.81 (R^2^ = 0.8650, P < 0.05), and Y = 1.6295X − 17.70 (R^2^ = 0.909, P < 0.05), respectively. The *in vitro* gas production method is useful for the determination of protein degradation in feedstuffs.

## Introduction

Protein supply is a key factor that determines the economic success of the beef cattle industry. Due to lack of information on the protein quality of beef cattle feedstuffs in China, the beef cattle industry relies on accurate analyses. The degradation rate of feedstuff protein in rumen is an essential characteristic for the determination of the protein value of feeds^1–3^ and an important feed characteristic in nutritional models^4–6^. Particularly, cereal grains such as maize, sorghum, barely and wheat which are rich in starch have long become an important component of the diet in high-producing beef cattle in China, and they also are a valuable source of proteins, while chemical structure and protein matrix leads to large variations in the protein degradation capabilities of the cereal grains^7^. Similarly, chinese wild rye grass, corn stalk, rice straw and soybean straw were important forages commonly used for beef cattle production in China especially districts of with less availability of feed resources due to their advantages of large-scale production and high nutritional value^8–10^. Besides, soybean meal, DDGS, brewers’ spent grains and sunflower meal are special source of protein for beef cattle feeds in China due to economic and environmental concerns, and these by-products usually have high protein contents and can provide competitive alternatives to more traditional protein sources^11^.

Amongst all methods used to evaluate protein quality in ruminant feedstuffs, *in vivo* estimates are the most reliable. Even though the *in vivo* method is expensive, time consuming, and laborious, it provides accurate information on the animal response to feedstuffs. In addition, data from *in vivo* studies are used to validate the accuracy of *in situ* and *in vitro* methodologies^12,13^. The *in vitro* gas production technique^14,15^ has been use to evaluate rumen CP degradability of feeds via ammonia-N and gas production measurements recorded by incubating feedstuffs in buffered rumen fluid. By improving this technique (e.g., through method innovation, instrument upgrades, and data handling), the *in vitro* gas production technique has been widely used to predict digestibility of ruminant feedstuffs^16–19^. Even though *in vivo* and *in vitro* methods have been widely researched, there are few comparisons between *in vitro* and *in vivo* measurements of feedstuff protein degradation.

In order to make the evaluation of relationship between IVPD and NPU more accurate, we choosed three types (forages, energy feed and protein feed) with large range of the content of protein. And the objectives of this study were to (1) determine the chemical composition of twelve common beef cattle feedstuffs commonly used in China and (2) predict protein digestibility by using *in vitro* gas production technique and *in vivo* data derived from digestion trials in an attempt to assess the accuracy of the *in vitro* gas production technique.

## Results

The chemical composition of twelve feedstuffs is shown in Table 1. Starch content in corn, sorghum, barley, and wheat was 66.69%, 52.40%, 55.14%, and 59.46%, respectively. Meals (soybean meal and sunflower meal) and by-products (DDGS and Brewers’ spent grain) contained more CP than cereals and roughages, less NDF and ADF than roughages, and less starch than cereals. Among the protein feed group, CP concentration was higher in soybean meal and sunflower meal than in DDGS and Brewers’ spent grain. NDF, ADF, and starch were higher in Brewers’ spent grain than in soybean meal, sunflower meal, and DDGS. Sunflower meal had the highest CF content among the protein feed group. The roughage feed group contained high levels of NDF and ADF. Soybean straw had higher CP than Chinese wild rye grass, corn stalk, and rice straw.

**Table 1:** Nutrient levels and composition of feedstuffs (%, DM).

	DM	CP	EE	Ash	CF	NDF	ADF	ADL	NDIN	ADIN	Starch
**Energy Feed (cereal)**											
Corn	85.14	10.85	4.12	1.83	2.05	11.61	4.03	1.14	0.14	0.06	66.69
Sorghum	86.25	17.92	3.23	3.41	3.00	13.69	7.56	2.02	0.44	0.43	52.40
Barley	87.30	16.37	3.54	2.95	4.37	19.70	7.35	1.98	0.31	0.08	55.14
Wheat	85.33	18.50	1.54	2.60	2.25	12.83	4.31	0.96	0.23	0.04	59.46
**Protein Feed (meal and by-products)**											
Soybean meal	87.09	47.77	3.21	6.41	7.35	28.19	10.46	2.22	0.38	0.16	6.04
DDGS	89.60	30.18	4.69	5.84	9.12	39.30	13.46	4.19	0.88	0.17	6.12
Brewers’ grain	91.46	28.79	4.83	7.72	25.28	56.12	32.05	8.24	0.50	0.17	13.10
Sunflower meal	79.79	40.79	0.70	7.54	29.29	37.98	27.44	9.67	0.46	0.21	3.26
**Roughage (straw feed)**											
Chinese wild rye grass	89.92	7.25	1.54	7.70	41.27	79.27	49.48	8.42	0.24	0.16	0.74
Soybean straw	88.46	12.75	1.08	8.26	51.24	72.14	56.01	13.70	0.30	0.51	1.01
Corn stalk	96.92	7.94	1.87	8.75	34.87	71.52	39.14	3.48	0.39	0.18	3.73
Rice straw	91.26	8.69	1.40	11.26	39.88	72.48	47.35	7.04	0.38	0.25	3.93

Protein intake, fecal protein, urinary protein, and NPU of the twelve feedstuffs are shown in Table 2. Protein intake consist of proteins provided by mixed feeds (energy feed group and protein feed group) or single feeds (roughage feed group). The type of feedstuff affected the amount of protein intake. Higher protein intake was observed in the protein feed group (1.9069 kg/d for soybean meal, 1.2455 kg/d for DDGS, 1.2198 kg/d for Brewers’ spent grains, and 0.9115 kg/d for sunflower meal), and lower protein intake was observed in the roughage feed group (0.3976 kg/d for Chinese wild rye grass, 0.6919 kg/d for soybean straw, 0.4322 kg/d for corn stalk, and 0.4779 kg/d for rice straw). Fecal protein was the highest for sorghum (0.3537 kg/d) and lowest for Chinese wild rye grass (0.1508 kg/d). Urinary protein was the highest for soybean meal (141.86 g/d) and lowest for corn stalk (18.19 g/d). NPU varied among the feedstuffs. In the protein feed group, the highest and lowest NPU values were obtained in soybean meal (82.41%) and Brewers’ spent grain (68.37%), respectively. Among the cereal grains, NPU was higher for wheat (72.58%) and barley (65.38%) than for corn (52.57%) and sorghum (49.68%). For roughages, soybean straw had the highest NPU (56.27%).

**Table 2:** Net protein utilization (%) of feedstuffs.

	Protein intake (kg/d)	Fecal protein (kg/d)	Urine protein (g/d)	Net protein utilization (%)
**Energy Feed (cereal)**				
Corn	0.5270	0.2182	29.12	52.57
Sorghum	0.7806	0.3537	33.02	49.68
Barley	0.7214	0.1919	45.19	65.38
Wheat	0.7867	0.1962	40.73	72.58
**Protein Feed (meal feed)**				
Soybean meal	1.9069	0.2265	141.86	82.41
DDGS	1.2455	0.2759	88.31	72.26
Brewers’ spent grain	1.2198	0.2186	89.44	68.57
Sunflower meal	0.9115	0.1985	116.56	76.95
**Roughage (straw feed)**				
Chinese wild rye grass	0.3976	0.1508	29.07	54.75
Soybean straw	0.6919	0.2603	42.26	56.27
Corn stalk	0.4322	0.2885	18.19	29.03
Rice straw	0.4779	0.2526	25.13	41.88

**Figure 1:**
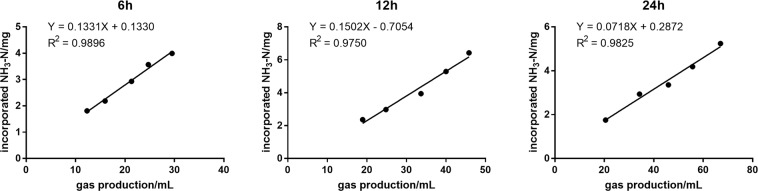
Regression equations of incorporated NH_3_-3 (y, mg) and gas production (x, mL) after 6, 12, and 24 h of incubation. Five point from left to right represent five levels of substrate that were prepared based on the amount of soluble starch (0 mg DM, 40 mg DM, 80 mg DM, 120 mg DM, and 160 mg DM).

Figure 1 shows a linear regression between NH_3_-N incorporated into microbial proteins and gas production after 6, 12, and 24 h of incubation. The linear regression was significant, indicating that gas production from feedstuffs is an accurate estimate of incorporated ammonia N. The highest correlation coefficient was observed at 6 h, Y = 0.1331X + 0.133, R^2^ = 0.9896 (P < 0.05). The linear regression equations at 12 and 24 h were Y = 0.1502X – 0.7054 (R^2^ = 0.9750, P < 0.05) and Y = 0.0718X + 0.2872 (R^2^ = 0.9825, P < 0.05), respectively.

Based on the linear regression equations, estimates of IVPD were calculated (Table 3). Protein degradation during the first 6 h of *in vitro* incubation varied from 68.43% (wheat) to 25.65% (sorghum). After 12 h of *in vitro* incubation, CP degradation ranged from 80.12% (wheat) to 36.6% (corn stalk). IVPD after 24 h of *in vitro* incubation was the highest for wheat (87.56%) and the lowest for corn stalk (44.42%).

**Table 3:** IVPD (%) of feedstuffs after 6, 12, and 24 h of incubation.

IVPD	Time		
	6 h	12 h	24 h
Corn	45.14	60.49	69.62
Sorghum	25.65	38.54	45.55
Barely	51.45	76.48	85.07
Wheat	68.43	80.12	87.56
Soybean meal	56.03	69.47	77.77
DDGS	47.24	51.29	66.71
Brewers’ spent grain	40.06	50.18	62.73
Sunflower meal	43.43	52.62	64.04
Chinese wild rye grass	43.17	51.09	79.96
Soybean straw	46.79	57.96	71.58
Corn stalk	31.48	36.6	44.42
Rice straw	33.65	50.56	72.59

**Figure 2:**
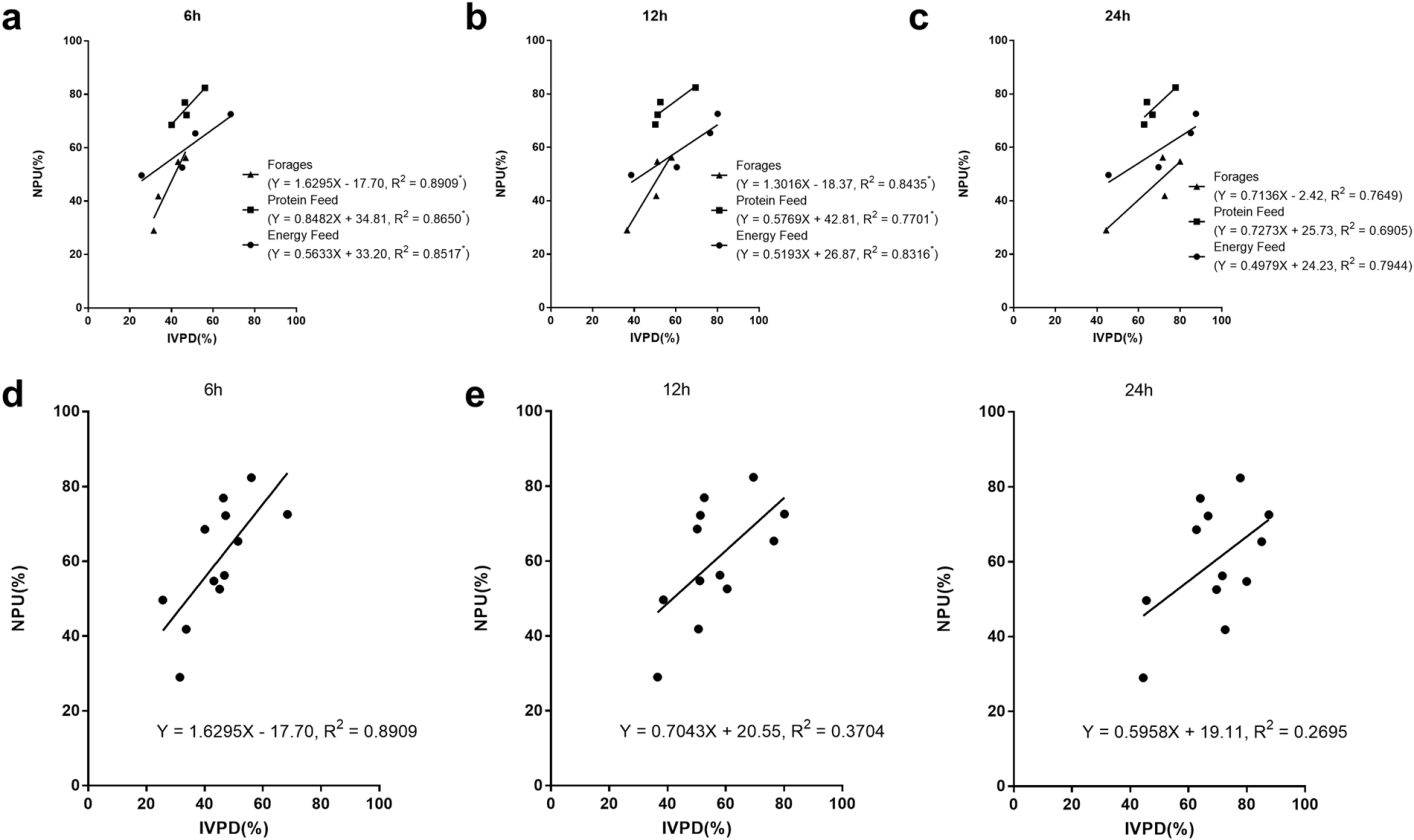
Linear equations between IVPD (x) and NPU among feedstuffs in the forages group, protein feed group and energy feed group at 6 h (**A**), 12 h (**B**) and 24 h (**C**), respectively. And linear equations between IVPD (x) and NPU among 12 feedstuffs at 6 h (**D**), 12 h (**E**) and 24 h (**F**).

The relationship between IVPD and NPU after 6, 12, and 24 h of incubation are presented in Fig. 2. Regression analyses demonstrated that the linear regression equations in the three feed groups (energy feed group, protein feed group, and roughage feed group) were significant (P < 0.05) after 6 h of incubation (R^2^ of 0.8517, 0.8650, and 0.8909, respectively). However, increased incubation time decreased the R^2^ value. At 12 h, the R^2^ values of IVPD and NPU in the energy feed group and roughage feed group were 0.8316 and 0.8435, respectively. Additionally, there was no correlation between IVPD and NPU in the protein feed group after 12 h of incubation. Similarly, no relationship was observed between IVPD and NPU in the energy feed group, protein feed group, and roughage feed group at 24 h. When IVPD and NPU were analyzed together, the linear regression equations at 6, 12, and 24 h of incubation revealed no correlation. The equations were described by Y = 0.9848X + 16.25 with R^2^ = 0.5171 and P > 0.05 (6 h of incubation), Y = 0.7043X+ 20.55 with R^2^ = 0.3704 and P > 0.05 (12 h of incubation), and Y = 0.5958X + 19.11 with R^2^ = 0.2695 and P > 0.05 (24 h of incubation).

## Discussion

The starch content of cereals (corn, sorghum, barley, and wheat) obtained in this study was lower than that reported by *Nutrient Requirements of Beef Cattle* (eighth revised edition, 2016), probably due to difference in cereal origin and growing conditions. Wheat and barley are two of the most readily degradable cereals due to their higher rate of ruminal starch fermentation and more extensive ruminal nitrogen fermentation compared with corn and sorghum^20,21^. The lower degradation rate of corn compared to that of wheat can be explained by the different nature of the protein components, particularly zeins in the former and gliadins in the latter^22^. The main potential limitation of sorghum is its poor digestibility due to the presence of dense proteinaceous matrix in the peripheral endosperm layer of the kernel, which renders protein inaccessible^23^. Soybean meal is a major contributor of protein to beef cattle. The CP, ADF, and NDF of soybean meal obtained in this study were similar to those reported by NRBC^6^. The utilization of feed protein in ruminants depends not only on total protein and amino acid content but also on the type of feed protein^24^ and its molecular structure^25^. Highest NPU values were observed in SBM, possibly due to the structure and solubility characteristics of SBM protein^26,27^. Brewers’ spent grain is a valuable protein source with higher levels of starch, NDF, and ADF than soybean meal, DDGS, and sunflower meal. Several studies have shown that Brewers’ spent grain, which consists of husks, pericarp parts, and seed coat layers of grains, is a rich source of fiber, protein, and carbohydrate^28,29^. In addition, early studies on sunflower meal have reported that it is high in fiber^30^ and a potential source of protein in beef rations^31^. Uniquely in beef cattle, the process of ruminal degradation of protein converts dietary protein of low biological value in low quality roughage into microbial protein of high biological value^32^. The CP content of soybean straw was higher than those reported by Maheri-Sis *et al*. and NRBC^6,33^. Soybean straw, which consists of stems, leaves, and pod husks, is a major source of roughage for beef cattle. Similar NPU values of soybean straw were reported by Gupta *et al*. (1978), who concluded that protein digestibility depends on variations in the pod-to-stem ratio and on maturity stage of harvested soybeans^34^. The presence of high levels of silica in rice straw may contribute to the resistance of rice straw to ruminal degradation^35^. The NPU of corn stalks and Chinese wild rye grass is low due to the formation of cross-links between lignin polymers and polysaccharides in the plant cell wall through phenolic acids (predominantly ferulic and p-coumaric acids) and arabinoxylans, that provide cell wall integrity and resistance against microbial enzymatic degradation^36,37^.

The approach we used in this study aimed to analyze the correlation between gas production and NH_3_-N incorporation into microbial proteins as an indicator of protein degradation. Gas production can be used to evaluate metabolisable energy content of feedstuffs^15^, which in turn determines the synthesis of rumen microbial protein. Dietary protein is eventually degraded into ammonia N in the rumen via enzymatic activity of ruminal microorganisms. However, ammonia from protein degradation and microbial catabolism, which occur simultaneously in the rumen, make it difficult to identify IVPD of feedstuffs from ammonia release. To eliminate the effect of ruminal microbial protein synthesis during fermentation, we used a method to eliminate the effect of ruminal microbial protein synthesis during fermentation. Raab *et al*. (1983) reported that the amount of NH_3_-N released when no fermentable carbohydrates are available and consequently no bacterial protein synthesis can take place can be represented by the intercept of the linear regression between NH_3_-N concentration and gas production. The results can be corrected by the difference between this intercept and NH_3_-N content in the blank (rumen fluid without substrate^14^. In our earlier studies, we attempted to determine protein degradability using techniques described by Broderick (1987) and Raab *et al*.^14,38^. However, the methods were complicated and cumbersome and had limitations on the batch culture used. In this study, we classified ruminal ammonia N into (1) incorporated ammonia N, which is used for microbial degradation of protein, and (2) free ammonia N. There was a high correlation between ammonia N incorporated into microbial proteins and gas production, suggesting that gas production of feedstuffs is an accurate estimate of incorporated ammonia N. Therefore, we hypothesize that free ammonia N and incorporated ammonia N represent the N obtained from feeds.

We obtained a significant correlation between IVDP and NPU of each type of feed; however, there was no correlation between IVDP and NPU when we conducted a unified analysis of all feeds. Different types of feedstuff differ substantially in their physical and chemical characteristics and are metabolized differently in animals. Falahatizow *et al*. (2015) reported that gas production rate might be related to structural carbohydrates of feedstuffs; consequently, gas production rate may affect IVDP^39^. Additionally, degradability is not only related to the type of feed but also to the residence time of feedstuffs in the rumen^14^. It is important to be aware of the retention time of feedstuffs to select the appropriate incubation time for the *in vitro* gas production technique. In the present study, IVDP increased with incubation time, and the highest correlation coefficient between feed protein degradation rate determined by *in vitro* gas production method and *in vivo* NPU was observed at 6 h of incubation in the three feed groups (energy, protein, and roughage). The *in vitro* gas production technique used in this study determined the end-point fermentability of feedstuffs by recording plunger displacement at frequent intervals. With increasing incubation time, IVDP increased and regression coefficient decreased. This observation is in agreement with the findings reported by Lorenz *et al*. (2011), who observed that increasing IVDP indicates higher NH_3_ released from protein degradation as opposed to bacterial uptake due to an energy shortage for bacterial synthesis^40^. Furthermore, with increasing *in vitro* culture time, fermentation products gradually accumulate due to the non-outflow of fermentation contents, thereby impacting the living conditions of microorganisms and fermentation. Eventually, these factors would be expected to interfere with normal microbial protein degradation. Mota *et al*. (2005) reported that underestimation of N degradation due to microbial synthesis that increases the proportion of N in the residue negatively affects the accuracy of the method^41^. Besides, the degradation degree of proteins contained in microorganisms increases with incubation time. Meng *et al*. (1991) reported that when the blank culture was performed *in vitro*, the concentration of free NH_3_-N increased gradually with time, and the concentration of NH_3_-N at 24 h was 1.54 times higher than the initial concentration of NH_3_-N^42^. Similarly, Cone *et al*. (1998) noted that microbial turnover in the blank sample resulted in a gas production rate that was different from that of feed samples and this could increase the ammonia concentration^43^. In addition, Karlsson *et al*. (2009) have speculated that different amounts of added carbohydrates affect the microbial turnover during longer incubation intervals. Pre-incubation with carbohydrates is essential for standardizing rumen fluid by increasing microbial activity and equilibrating the initial ammonia (NH_3_) concentration^44^. In previous experiments designed to study a optimum carbohydrate source, we observed that artificial rumen fluid with 25 mg of soluble starch and 25 mg of maltose was optimum^42^. However, the factor that corrects rumen fluid may be different for different feedstuffs, resulting in different regression coefficients. Therefore, it is important to set up a regression equation by choosing the feed itself as the nitrogen source and adding the starch by grades.

The *in vivo* method is the gold standard for determining ruminal CP degradability. The weak correlation between the *in vivo* and *in vitro* methods is due to variations in the latter. The quality of rumen fluid has a great impact on *in vitro* gas production technology. Our previous studies found that different sources of rumen fluid and sampling time had no effect on IVPD. However, in this study, feedstuffs were not incubated in defaunated rumen fluid. Ignoring the presence of protozoa in rumen fluid may influence *in vitro* measurements of feedstuff. Lorenz *et al*. (2011) reported that when soybean meal was incubated as a protein source, the removal of protozoa *in vitro* by centrifugation minimized the recirculation of bacterial protein by protozoa^40^. However, Rymer *et al*. (2005) observed that rumen fluid continuously flushed with CO_2_ during incubation may adversely affect gas production by excluding adherent cellulolytic bacteria from the rumen inoculum^45^. Furthermore, protein degradability has been generally estimated at the end-point of incubation, which provides inadequate information of the degradation pattern over time. To compare the ruminal degradability of feedstuffs, we need to obtain kinetic parameters, which could be offered by modified gas production instruments. Pellikaan *et al*. (2011) and Elberg *et al*. (2018) modified the *in-vitro* gas production instrument and reported total gas production and a precise description of *in-vitro* gas kinetics^44,46^. There is a need to generate a standardized approach of the *in vitro* gas production technique.

In summary, there were significant correlation between IVPD and NPU in the same feed type, and *in vitro* gas production method could be used for rapid determination of protein digestibility. More samples are needed for building more accurate relationship between IVPD and NPU in different feed type prediction for further study.

## Methods

### Feed samples

Twelve feedstuffs were obtained from Beijing (Hebei Province, P. R. China), air-dried, and ground in a hammer mill to pass through a 2-mm sieve for chemical analysis. In the *in vivo* method, the feedstuffs were divided into three groups: (1) energy feed group (cereals): corn, sorghum, barley, and wheat; (2) roughage feed group: Chinese wild rye grass, corn stalk, rice straw, and soybean straw; (3) protein feed group (meal and by-products): soybean meal, distillers’ dried grains with soluble (DDGS), Brewers’ spent grains, and sunflower meal.

### Chemical analysis

Feeds and animal feces were analyzed for dry matter (DM), ash, and ether extract (EE) according to AOAC (2012) methods. Crude protein (CP), acid detergent insoluble N (ADIN), and neutral detergent insoluble N (NDIN) were determined by the Dumas method using an N Analyzer (Rapid N III, Elementar, Germany) and a nitrogen-to-protein conversion factor of 6.25. Neutral detergent fiber (NDF), acid detergent fiber (ADF), and acid detergent lignin (ADL) were analyzed using an A220 Fiber Analyzer (ANKOM Technology Corp., Macedon, NY, USA). NDF was assayed using alpha-amylase. Both NDF and ADF contained no residual ash. Urinary N was analyzed according to AOAC (2012) methods.

### *In-vitro* Gas production method

Rumen fluid was collected from three Angus steers (approximately 320 kg body weight) fitted with permanent rumen cannulas. The animals were fed a total mixed ration which consisted of 40% steam-flaked corn, 40% corn stalk silage, 4.5% cottonseed meal, and 13.5% brewer’s grains (DM basis) and 0.5% salt and 0.5% premix (2.4 g/kg Mg, 7.6 g/kg K, 12.0 g/kg Fe, 1.0 g/kg Cu, 200 mg/kg Mn, 650 mg/kg Zn, 2 mg/kg Se, 22 mg/kg I, 9 mg/kg Co, 121,000 IU/kg vitamin A, 37,400 IU/kg vitamin D, and 55 IU/kg vitamin E) twice a day with *ad libitum* access to water. Animal care and use were approved and conducted according to standards established by the College of Animal Science and Technology, CAU, Beijing, P. R. China (permit number DK1402006). *In vitro* incubation was carried out according to the procedure reported by Menke *et al*. (1979). Samples of rumen fluid were collected prior to the morning feeding, passed through four layers of cheesecloth into a pre-warmed vacuum bottle, and transported immediately to the laboratory of Beef Cattle Research Center of China Agricultural University. The rumen fluid was mixed with buffer solution in a 1:1 (v/v) ratio (Menke *et al*., 1979) under a continuous flux of CO_2_ to maintain anaerobic conditions and maintained at 39 °C.

Five levels of substrate were prepared based on the amount of soluble starch: 0 mg DM, 40 mg DM, 80 mg DM, 120 mg DM, and 160 mg DM. An equal amount of (NH_4_)_2_SO_4_ (10 mg N) was added to a glass syringe. Artificial rumen culture solution (30 mL, containing 25 mg of soluble starch and 25 mg of maltose) was added and incubated at 39 °C. Gas production was measured at 6, 12, and 24 h incubation. The syringe was transferred to an ice bath to prevent further microbial activity. The fermentation mixture was centrifuged at 1,000 g for 10 min. The resulting supernatant was used for the determination of incorporated NH_3_-N.

The feed samples in each syringe contained 200 mg DM and <10 mg N. If the N content of the sample was >5%, the additional supplementation of corn starch would result in a DM content of 200 mg; conversely, if the N content of the feed sample was <5%, only 200 mg of this sample needed to be weighed separately. Incubations were performed in triplicate. Blank samples consisted of three syringes with artificial rumen culture solution.

### *In vivo* Method

Twelve Angus steers (320 ± 20 kg body weight) fitted with permanent rumen cannulas (CAU Beef Cattle Research Center, Beijing, P. R. China) were kept individually in cages. The animals had *ad libitum* access to water and were assigned to one of three groups (each group consisted of four steers) based on their body weight in a 4 × 4 Latin square design. An adaptation period (15 d) was followed by a sample collection period (5 d). During the collection period, fecal and urine samples were collected after the morning feeding. Animal care and use were approved and conducted according to standards established by the College of Animal Science and Technology, CAU, Beijing, P. R. China (permit number DK1402006).

The steers were fed the experimental diets at 1.9% BW on a DM basis in two equal portions at 08.00 and 16.00. To prevent the occurrence of digestive abnormalities such as rumen acidosis by feeding a single feed, the first group (energy feed group) and the third group (protein feed group) were fed a mixed diet of 4.2 kg of feed and 1.8 kg of Chinese wild rye grass; the second group (roughage feed group) was fed the single feed. To meet nutrient balance, 2% rumen buffer (NaHCO_3_:MgO = 2:1), 0.5% stone powder, and 0.5% salt of feed intake on a DM basis and 30 mg/kg Rumensin was added to ensure normal ruminal fermentation.

Fecal samples were collected on sampling days, and 20% of the total fecal weight was collected. Fecal samples were collected into bags containing 10% tartaric acid to prevent the loss of ammonia nitrogen (N) and stored at −20 °C. At the end of each collection period, fecal samples were pooled and weighed. The pooled fecal samples were naturally air-dried, ground to a 1-mm screen size, and stored at 0–4 °C. Urine samples were collected in urinary bags. The bag, which was designed by a research team at the China Agricultural University (CAU; Beijing; P. R. China), consists of a funnel, a catheter, and latex tubes. Urine volume was measured daily, and the urine samples were passed through a gauze. The urine samples were collected in 600 mL of 6 N HCl to prevent the loss of ammonia nitrogen (N) and stored at 4 °C.

### Statistical analysis

IVPD was estimated for each feed after each incubation period in the GP experiment via linear regression of incorporated ammonia N (*y*, mg) vs. gas production (*x*, mL) (Meng *et al*.). The relationship between ammonia N incorporated into microbial proteins and gas production was quantitatively reflected by the regression coefficient *b*. IVPD was calculated at 6, 12, and 24 h using the following equation,

$${\rm{IVPD}}=\frac{({\rm{GP}}-\mathrm{GP}\,\,\mathrm{of}\,\,{\rm{blank}})\ast b+{\rm{free}}\,{\rm{ammonia}}-{\rm{N}}}{\mathrm{total}\,\,{\rm{N}}}\ast 100 \% $$

Data obtained from the *in vitro* and *in vivo* methods were statistically analyzed using EXCEL while taking into account the net protein utilization (NPU) of the four roughage samples (Chinese wild rye grass, soybean straw, corn stalk, and rice straw) according to the following statistical model,

$${\rm{NPU}}=\frac{(\mathrm{Intake}\,\,\mathrm{CP}\,-\mathrm{Fecal}\,\,\mathrm{CP}\,-{\rm{Urine}}\,{\rm{CP}})}{(\mathrm{Intake}\,\,\mathrm{CP}\,-{\rm{Fecal}}\,{\rm{CP}})}\times \frac{(\mathrm{Intake}\,\,{\rm{CP}}-{\rm{Fecal}}\,{\rm{CP}})}{{\rm{Intake}}\,{\rm{CP}}}$$

Protein degradation data obtained from corn, sorghum, barley, wheat, soybean meal, DDGS, Brewers’ spent grain, and sunflower meal were analyzed according to the concentration of CP in Chinese wild rye grass using the follow equation,

$$\begin{array}{c}{\rm{NPU}}=\frac{({\rm{Intake}}\,{\rm{CP}}-{\rm{Fecal}}\,{\rm{CP}}-{\rm{Urine}}\,{\rm{CP}}+\mathrm{Chinese}\,\,{\rm{wildrye}}\,{\rm{grass}}\,{\rm{CP}}\times (1-{\rm{Chinese}}\,{\rm{wildrye}}\,{\rm{grass}}\,\mathrm{CP} \% ))}{({\rm{Intake}}\,{\rm{CP}}-\mathrm{Fecal}\,\,{\rm{CP}})}\\ \,\times \frac{({\rm{Intake}}\,{\rm{CP}}-{\rm{Fecal}}\,{\rm{CP}})}{\mathrm{Intake}\,\,{\rm{CP}}}\end{array}$$The regression analysis between the two methods was determined by the CORR and REG procedures of SAS(version 9.4). Duncan’s significant difference test procedure was used to determine differences among means. Significance was set at P ≤ 0.05.
